# Genetic Diversity of *Brucella melitensis* Isolated from Domestic Ruminants in Iraq

**DOI:** 10.3390/microorganisms12030475

**Published:** 2024-02-27

**Authors:** Fabrizio De Massis, Ruqaya Mustafa Ali, Sara Serrani, Michela Toro, Alessandra Sferrella, Nausica D’Aurelio, Anna Janowicz, Katiuscia Zilli, Teresa Romualdi, Eugenio Felicioni, Manhal Habeeb Salman, Dunya Hatem Fahdel, Hiba Saad Rashid, Bilal Qays Ameen, Giuliano Garofolo

**Affiliations:** 1 WOAH Reference Laboratory for Brucellosis, Istituto Zooprofilattico Sperimentale dell’Abruzzo e del Molise “G. Caporale”, 64100 Teramo, Italy; f.demassis@izs.it (F.D.M.); m.toro@izs.it (M.T.); a.sferrella@izs.it (A.S.); n.daurelio@izs.it (N.D.); k.zilli@izs.it (K.Z.); t.romualdi@izs.it (T.R.); e.felicioni@izs.it (E.F.); g.garofolo@izs.it (G.G.); 2 Ministry of Agriculture, Veterinary Directorate, Central Veterinary Laboratory (CVL), Baghdad 8CV3+4XQ, Iraq; rukia_mustafa19@yahoo.com (R.M.A.); kawkabalfityan@yahoo.fr (M.H.S.); doniaaltaee55@gmail.com (D.H.F.); hayash809@gmail.com (H.S.R.); bilalqais799@yahoo.com (B.Q.A.)

**Keywords:** *Brucella melitensis*, Iraq, MLVA-16, MLST, WGS, epidemiology

## Abstract

The control and eradication of brucellosis represents a critical objective for Veterinary and Health Authorities across several countries globally. Efficient surveillance programs play a pivotal role in detecting and managing outbreaks. Epidemiological investigations significantly benefit from standardized and efficient molecular typing techniques and analytical tools, enabling public health laboratories to identify the origin of outbreaks. This study aimed to sequence *Brucella* spp. strains isolated in Iraq from different ruminant species to verify their molecular epidemiological correlations and, above all, to shed a light on how these Iraqi isolates are positioned in the phylogenetic context of *Brucella* spp. The 35 isolates under study were from abortion, milk, placenta, and the fetal membranes of sheep, cattle, and buffalo. Genotyping involved various techniques: MLVA-16, Whole Genome Sequencing, MLST, and cgMLST. All the Iraqi isolates from our study clustered in MLVA-16 within the East Mediterranean clade, and all but one grouped together in the same branch of the MST tree. MST analysis showed the minimum distance of one allele between the studied isolates, except for one strain from buffalo, which was positioned farther away from the rest of the isolates. In cgMLST, the majority of strains grouped within a large cluster predominantly comprising genotypes from the Middle East. The application of different control measures in different territories based on molecular epidemiological studies would increase the chances of maximizing public health benefits and minimizing the spread of infection to disease-free or lower prevalence areas.

## 1. Introduction

Human brucellosis is still a current problem. In the world, the epidemiological situation is not understandable, for example, control programs [1]. Sekhi [2] emphasized the significance of protecting individuals, as they can also contract the disease through the inhalation of contaminated dust or aerosols. Furthermore, Sekhi [2] reported a notable rise in the prevalence of both acute and chronic brucellosis in Iraq.

People may also be infected by the inhalation of contaminated dust or aerosols; therefore, *Brucella* species have been labeled as highly weaponizable [2].

The effective control of animal brucellosis involves three crucial steps: surveillance, the prevention of transmission, and eradication from the reservoirs. Limiting the spread of the disease necessitates strict control over the movement of infected animals, particularly those from epidemic areas [3].

In the Middle East countries, there is underreporting and insufficient monitoring data about brucellosis, so the incidence is not truly detected. Consequently, the disease continues to exhibit sustained prevalence [4].

In low and middle-income countries, misconceptions about the true incidence of *Brucella* often arise from underreporting and inadequate monitoring data. A lack of financial resources and capacity, along with limited collaboration between veterinarians and human medics, further contributes to the sustained prevalence of brucellosis [4]. Paired with the presence of rampant social stigmas and mistrust regarding government intervention, this has allowed *Brucella* to increase unchallenged in the marginalized human and animal populations of the world.

Brucellosis is also a growing global One-Health issue due to the increase in international commodity exchange, which allows the infection to spread from endemic areas to other parts of the world [5].

Brucellosis is transmitted through direct contact with infected sheep or goats and by consuming unpasteurized milk and dairy products contaminated with the bacteria [6]. The highest incidence of brucellosis was documented within a sizable flock, and this was attributed to the demanding nature of maintaining hygienic conditions within these flocks. Routine cleaning and disinfection of the paddock, involving the removal of manure and other unsanitary waste, necessitates diligent effort [7]. Clinical manifestations of brucellosis are flexible and consist of fever and localized inflammatory processes [8]. In humans, the disease can affect many organ systems, and the most frequently reported symptoms include pyrexia, arthritis, and fatigue [4]. If the disease persists for days or months, it may evolve into a chronic form [7].

Over the past two decades, studies carried out in Iraq have reported a notable increase in the prevalence of human brucellosis [3]. Previous serological surveillance studies revealed the prevalence of the disease in small ruminants, cattle, buffalo, and camels as 6.51%, 1%, 1.84%, and 0.02%, respectively. The incidence rate of brucellosis in humans following the 2003 war was 5347 recorded cases, representing 22.7 cases/100,000 inhabitants [9]. The eradication of brucellosis in humans depends upon the eradication of brucellosis in susceptible animal species [10] and, in this framework, the isolation of *Brucella* strains circulating in a territory and their characterization from the genomic point of view is of utmost importance for the assessment of the epidemiological situation.

When compared to traditional microbiological or serological tests, the molecular approach for *Brucella* genomes has demonstrated the highest resolution [11].

Multiple-Locus Variable-Number Tandem Repeat (VNTR) Analysis (MLVA) has been in the past the most used molecular typing method [8].

To uncover more genetic features and facilitate comparisons between strains, Whole Genome Sequencing (WGS) -based phylogeny stands as the most powerful tool to discriminate *Brucella* strains and to investigate epidemiological spreading risk factors, through the analysis of their phylogenetic relationships. This approach has gained significant traction in *Brucella* research due to the increasing availability of *Brucella* genomes in publicly accessible databases [12]. This approach would improve the understanding of the risk factors for the persistence and spread of the infection, thus allowing decision makers to implement the appropriate corrective measures on the control strategies in place, thereby reducing the disease burden in animals and, subsequently, in humans.

Efficient surveillance programs are crucial in detecting and managing outbreaks, and they rely on the collection and accessibility of sound epidemiological data. Epidemiological investigations can benefit significantly from the presence of standardized and efficient molecular typing techniques and analytical tools. These tools enable public health laboratories to identify the origin of an outbreak, enhancing the precision and effectiveness of the response measures [13].

Regarding the genomic characterization of strains, the discriminatory power of a single locus-specific sequence analysis is generally limited as it involves only a limited region of the genome. In the *Brucella* genus, phylogenetic markers such as 16S rRNA genes are highly conserved with an almost 100% identity. Therefore, molecular methods such as Multilocus Sequence Typing (MLST), Multiple Loci VNTR (Variable Number of Tandem Repeats) Analysis (MLVA), and, more recently, Whole Genome Sequencing (WGS) typing methods are additionally used to discriminate between *Brucella* strains. These methods offer higher-resolution genetic clustering, enabling more precise identification of the source of outbreak cases [14,15].

The objective of this study was to sequence *Brucella* spp. strains isolated in Iraq from various domestic ruminant species to verify their molecular epidemiological correlations and, above all, investigate how these Iraqi isolates are positioned in the phylogenetic context of *Brucella* spp.

## 2. Materials and Methods

**Figure 1:**
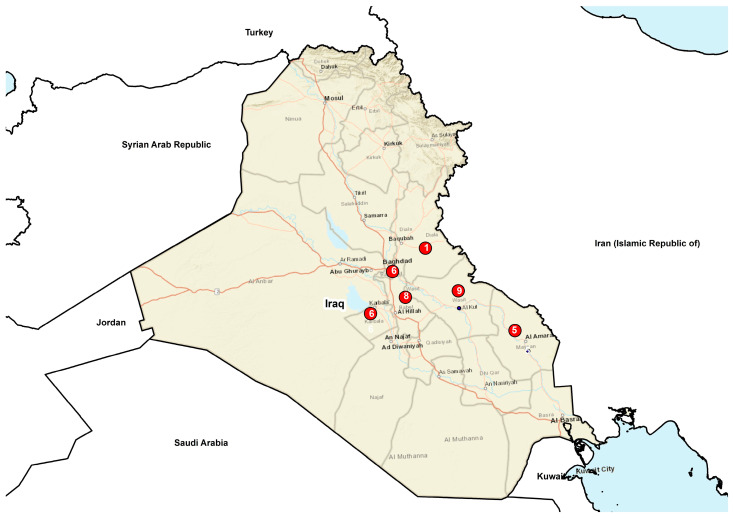
Distribution of *B. melitensis* strains collected from ruminants in Iraq in the 2015–2017 period. From each of the six governorates investigated, the figure indicates the number of *B. melitensis* strains isolated.

The strains under study (n = 35) were isolated from abortion, milk, placenta, and the fetal membrane of sheep (n = 22), cattle (n = 12), and buffalo (n = 1) in different governorates of Iraq (Figure 1). They were cultured in the Center for Brucellosis and Tuberculosis Control in the Iraq CVL and were isolated during the 2015–2017 period. The DNA was extracted by means of a QUIAamp Mini Kit (Qiagen) according to the manufacturer’s instructions and was identified as *B. melitensis* by real-time and conventional PCR methods in the WOAH Reference Laboratory for Brucellosis, Istituto Zooprofilattico Sperimentale dell’Abruzzo e del Molise “G. Caporale”, Teramo, Italy. Overall, the data on the isolates collected are summarized in (Table 1).

### 2.1. Multiple-Locus Variable-Number Tandem Repeat Analysis (MLVA)

The MLVA-16 samples were genotyped using the MLVA-16 panel described by Le Flèche et al. [16].

The DNA extracted from each *B. melitensis* isolate was amplified by multiplex PCR using primers specific for 16 MLVA loci to assign specific MLVA-16 alleles, as previously described by Garofolo et al. and Le Flèche et al. [16,17]. The amplified fragments were separated by capillary electrophoresis using an ABI 3500 Genetic Analyzer with POP-7TM Polymer, and the allele types were assigned using Genemapper 4.1 (Applied Biosystems, Carlsbad, CA, USA).

The MLVA-16 profiles were analyzed using the goeBURST algorithm implemented in PHYLOViZ, version 2.0. Minimum spanning trees (MST) were created using the default software settings [18].

### 2.2. Whole Genome Sequencing

The total genomic DNA was quantified using a Qubit fluorometer (QubitTM DNA HS assay; Life Technologies, Thermo Fisher Scientific Inc., Waltham, MA, USA). A Nextera XT library preparation kit (Illumina Inc., San Diego, CA, USA) was used to prepare the sequencing libraries according to the manufacturer’s protocol and the libraries were sequenced using the Illumina NextSeq 500 instrument. The sequencing produced 150 bp paired-end sequencing reads that were demultiplexed and, subsequently, the adapters were removed. The reads were trimmed from the 5′ and 3′ ends using Trimmomatic version 0.36 to discard the nucleotides with quality scores of less than 25, and the reads shorter than 36 bp were removed. The trimmed reads were de novo assembled using SPAdes version 3.11.1 with the careful option selected. Contigs shorter than 200 bp were removed from the draft genomes.

### 2.3. MLST and cgMLST

Ridom SeqSphere+, v6.0.2 (Ridom GmbH, Münster, Germany) was used to genotype the strains using the MLST and cgMLST schemes available through the software. MLST profiles were assigned using the *Brucella* 9 locus Multilocus Sequence Typing (MLST-9) scheme (https://pubmlst.org/brucella/ accessed on 9 September 2022) and cgMLST profiles were calculated using the *B. melitensis* task template with 2704 target core genes, as described by Janowicz et al. in 2018 [19]. Sequences containing less than 98% of cgMLST good targets were removed and a Multiple Spanning Tree (MST) was generated by pairwise comparison of the target genes using the default parameters. Missing values were ignored in the calculation of the distance between the pairs of sample profiles.

In the cgMLST analysis, we additionally included 105 genomes available in GenBank (with known geographical origin) and assigned to the East Mediterranean clade by Janowicz et al. [20] and 28 strains with known origin grouped in the East Mediterranean clade published in Sacchini et al. [15].

## 3. Results

### 3.1. MLVA Analysis

The strains were genotyped using MLVA-16 and twelve different MLVA profiles were assigned, with a maximum distance of two alleles between individual genotypes. Fourteen loci were identical between all the isolates and the variation occurred only in Bruce04 and Bruce16 (Table 2).

**Figure 2:**
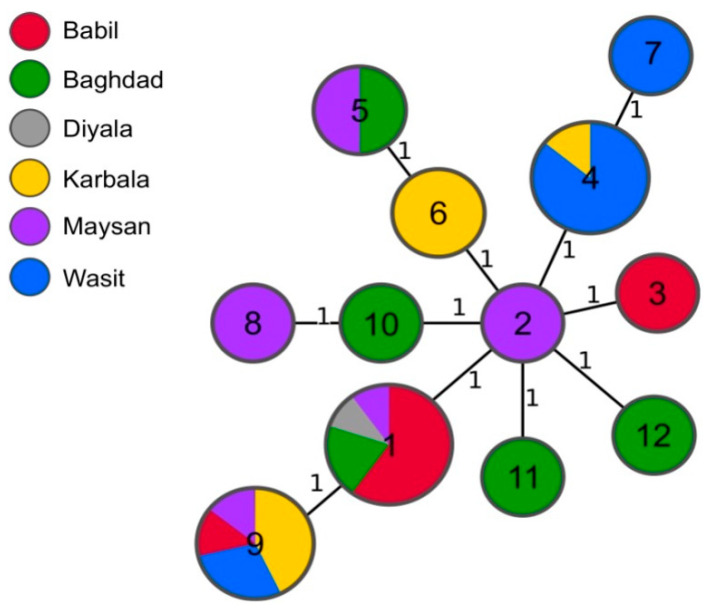
Minimum spanning tree of 35 Iraqi *B. melitensis* strains based on their MLVA-16 results. Nodes represent MLVA-16 profiles and are colored according to the governorate where the samples were isolated. Node labels correspond to the MLVA-16 profile number. The numbers on the branches correspond to the allele differences between genotypes.

The most common profile (1-5-3-13-3-2-3-2-4-40-8-4-4-3-15-4) was shared by ten strains isolated from sheep and cattle in four different governorates: Babil, Baghdad, Diyala, and Maysan (Table 1, Table 2). The second most frequently assigned profiles were 1-5-3-13-3-2-3-2-4-40-8-8-4-3-15-4, found in strains from Babil, Karbala, Maysan, and Wasit, and 1-5-3-13-3-2-3-2-4-40-8-4-4-3-19-4, from Karbala and Wasit. Both profiles were found in strains isolated from cattle and sheep. The minimum spanning tree of 35 Iraqi *B. melitensis* isolates based on their MLVA-16 results is shown in Figure 2.

The comparison of the MLVA-16 profiles of the strains analyzed in this study with the profiles obtained from the public database did not reveal any shared genotypes. Notably, all the Iraqi isolates from our study formed a distinct cluster within the East Mediterranean clade. With the exception of one strain, all the isolates clustered together in the same branch of the MST tree.

**Figure 3:**
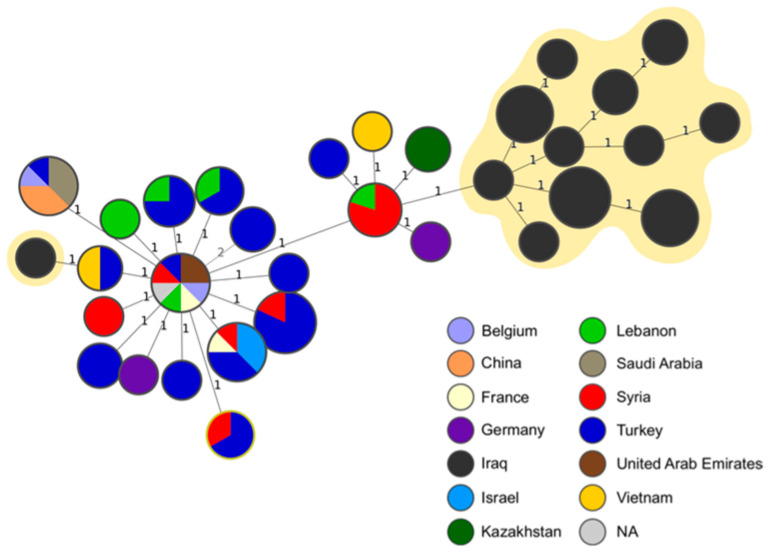
Minimum spanning tree (MST) of *B. melitensis* based on MLVA-16 results. The MST fragment shows specific connections of the Iraqi strains with the genotypes present in the public MLVA database. The nodes represent MLVA-16 profiles and are colored according to the country of origin of the analyzed strains. The numbers on the branches correspond to the allele differences between genotypes. The strains sequenced in this study are highlighted in yellow.

The MST analysis revealed a minimum distance of one allele between our isolates and other strains, with the most frequent connection observed in the MLVA profiles assigned to the isolates originating in Syria (Figure 3). Notably, one strain obtained from buffalo was positioned farther away from the rest of the isolates from our study and showed a connection to strains from Turkey and Vietnam (Figure 3).

### 3.2. Whole Genome Sequencing

**Figure 4:**
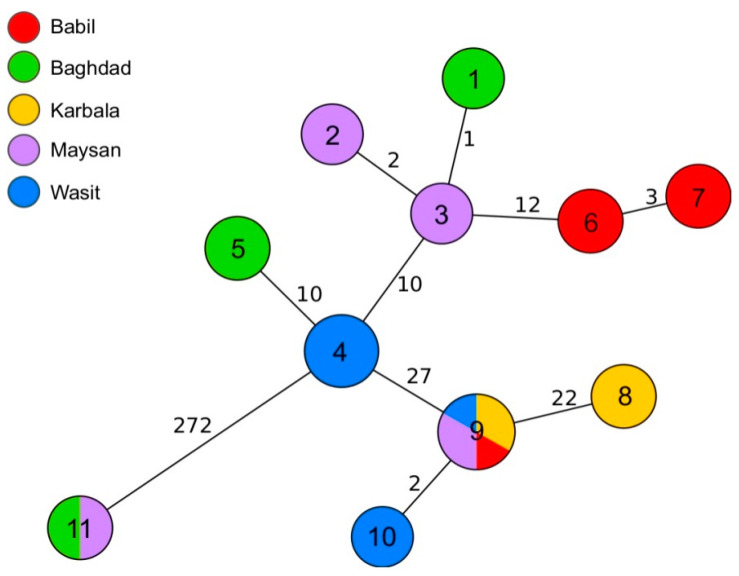
Minimum spanning tree (MST) of 35 Iraqi *B. melitensis* strains based on their cgMLST results. MST was calculated by pairwise comparison of 2704 target genes with missing values ignored. The nodes represent cgMLST profiles and are colored according to the governorate where the samples were isolated. The nodes are labelled with the cgMLST profile number. The numbers on the branches correspond to the allele differences between genotypes.

Out of 35 samples analyzed by WGS, 10 passed the cgMLST quality threshold and were assigned cgMLST profiles (Table 2). The majority of the sequences grouped together within a 35-allele distance (Figure 4). Several more closely related clusters were also observed. Out of these, the biggest, which contained the cgMLST profiles 9 and 10, was assigned to isolates from four governorates: Babil, Karbala, Maysan, and Wasit. Six strains from these clusters shared the same MLVA-16 profiles. The second biggest cluster comprised grouped strains with the cgMLST profile 4, all of which were collected in Wasit. Additionally, no more than three alleles of difference were observed between profiles 1, 2, and 3 from Baghdad and Maysan and profiles 6 and 7 from Babil. Interestingly, two strains collected from sheep in Baghdad and Maysan were distant by 272 alleles from all the other genotypes. A similar relation was not identified by MLVA-16 typing (Figure 4).

### 3.3. MLST and cgMLST

**Figure 5:**
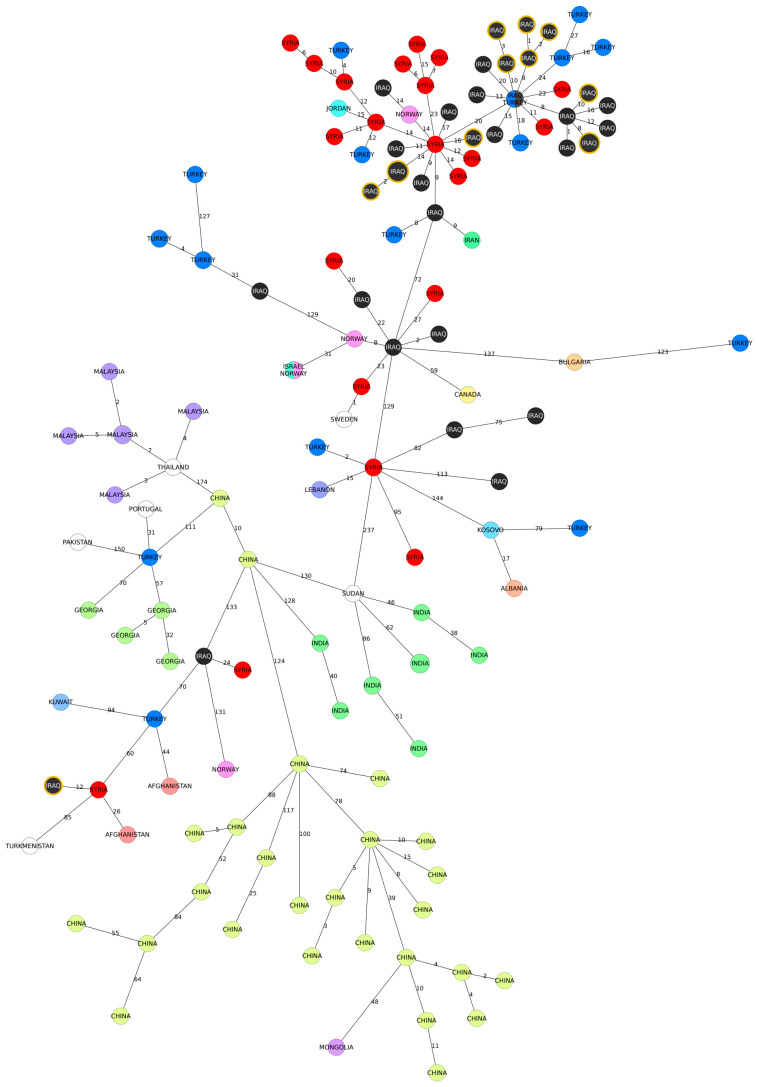
Minimum spanning tree (MST) generated for 158 isolates of *B. melitensis* using the cgMLST approach. MST was calculated by pairwise comparison of 2704 target genes with missing values ignored. The nodes represent cgMLST profiles and are colored and labeled with the country of isolation. The branch labels correspond to the allele differences between genotypes. Iraqi strains sequenced in this study are highlighted in black with a yellow circle.

Then, we compared the strains from our study to publicly available sequences from the East Mediterranean clade. The majority of the strains was grouped within a large cluster predominantly comprising genotypes from the Middle East (Figure 5). The minimum distance between our sequences and other available genotypes was eight alleles, and the closest related genomes included sequences from Iraq, Syria, and Turkey. The two Iraqi strains that were previously shown to be distant by 272 alleles from the other strains from the dataset were positioned in a separate branch of the MST containing few genotypes. The closest neighboring strain originating in Syria differed by 12 cgMLST alleles from the two strains from our dataset. The raw sequencing data generated in this study are available under NCBI Bioproject accession no. PRJNA1070976.

## 4. Discussion

Brucellosis continues to be endemic for various reasons, including the lack of veterinary support services and vaccines, the expansion of livestock herds and flocks, and husbandry practices that favor the spread of infection [21].

In Iraq, brucellosis holds significant importance as one of the most important zoonotic diseases, with confirmed cases dating back to 1937. Decades of unstable socio-economic and security-political conditions, coupled with the presence of the disease in livestock, have led to significant increases in human infections. There was a significant increase in disease incidence during the1990s due to the United Nations (UN) sanction, particularly in 1995 when brucellosis reached 88.5 cases/100,000 people according to the official records of the Ministry of Health [22].

Approximately 12 million Iraqis reside in rural areas and depend on agriculture for their livelihoods. Cattle, sheep, and goats are farmed for the production of meat, wool, milk, and leather. In Iraq, after agricultural production, livestock is the second largest sector providing income to the general population. The years of conflict in Iraq have resulted in the destruction of or severe damage to crops, equipment, livestock, seeds, and food reserves, leaving 3.2 million Iraqis food insecure [23].

Several Middle Eastern and central Asian countries have recently reported an increase in the incidence of human brucellosis and the appearance of new foci [4]. Among the Middle East countries, Syria, Saudi Arabia, Iraq, Iran, and Turkey have reported the highest annual incidence rates of human brucellosis worldwide as 160, 21, 28, 24, and 26 cases/100,000 persons/year, respectively [24].

Brucellosis is one of the most contagious epidemic diseases in ruminants, particularly in Iraq where the disease poses a substantial threat to both animal and human populations [25]. The presence of the infection at elevated levels across various species underscores the imperative need for the enhancement of control programs. Furthermore, different *Brucella* species may play a significant role in the transmission of brucellosis to humans, especially *B. melitensis*, which spreads through unpasteurized milk or associated dairy products [26].

The spread of brucellosis in Iraq can also be linked to travel activities, as individuals can carry the disease from endemic areas to different destinations, thereby posing a risk of dissemination [27].

PCR-based MLVA is the most commonly used approach for outbreak investigations and is still considered the gold standard for *Brucella* typing [28].

The genotypes described in this study have been found in neighboring governorates but also in distantly related regions. The presence of these genotypes in neighboring areas can be attributed to similar breeding practices that are prevalent across the region, suggesting a relatively consistent epidemiological situation of brucellosis in adjacent governorates. However, the study also identified distantly related genotypes, some of which were related to strains isolated in foreign countries, such as Syria and Lebanon (with one allele difference in MLVA-16) (Figure 3) or Turkey, Vietnam, and Kazakhstan (with two alleles difference in MLVA-16) (Figure 3). The Northern provinces of Iraq share an extensive border with Iran, Turkey, and Syria. Other provinces of Iraq share borders with Jordan, Saudi Arabia, and Kuwait. This geographic situation highlights the need for the strategic planning of control measures.

A similar study conducted in Europe (Portugal, Spain, Germany, Hungary, and Belgium) utilizing MLVA-16 genotyping and WGS methods identified *B. melitensis* as the major target in animals and humans [28].

In a genomic study focused on yaks in Tibet, Sun et al. employed the same methodology (MLVA-16, MLST, and WGS) and observed a prevalence of *B. abortus*. Interestingly, the strains originated from European countries [13], highlighting the significance of controlling animal movement as a pivotal point.

This underscores the need to investigate the international connections of Iraqi strains to identify potential risk factors for the introduction of the disease. Such an investigation should encompass examining the relationships with neighboring countries like Turkey and Syria (possibly due to infections from shared pastures) or more distant nations (likely associated with the trade of live animals).

One of the strengths of this study lies in the analysis of a database containing data on *Brucella* spp. isolated from animals, which provides added value compared to studies that rely solely on the sequences of isolates coming from returning travelers. This approach allows for a more direct and comprehensive understanding of the local situation regarding animal brucellosis in Iraq. However, a limitation of this study could be the relatively small dataset available, emphasizing the importance and need for larger and more robust datasets for more accurate future research. It is essential to acknowledge that such an approach can be resource-intensive, demanding significant financial resources [29].

Variable-Number Tandem Multiple Repeat Analysis (MLVA) and Whole Genome Sequencing (WGS) are used to analyze different parts of the genome and reflect different evolutionary processes; therefore, they can have different performance as tools for the surveillance and epidemiology of *Brucella*.

Both methods offer greater discrimination than other typing schemes, and discriminating power is one of the most important selection criteria for a genotyping method. However, it is important to consider that the discriminating capacity of a method is influenced by other factors, such as the enzymes and primers used, the enzymatic and amplification conditions, and the epidemiological characteristics of the microorganisms being tested.

Furthermore, the choice of genotyping method should also consider the epidemiological context under study; the surveillance of “local epidemic events” of bacterial infections can only be carried out using rapidly evolving markers, such as those studied using MLVA. On the other hand, for population or long-term epidemiological studies, methods based on the investigation of stable and conserved markers, such as Whole Genome Sequencing, are preferred.

Due to the broad and nonspecific consequences of brucellosis, the actual disease burden often goes undiagnosed, leading to a lack of prioritization of brucellosis control in the context of national control plans for animal diseases and zoonosis prevention [30]. The involvement of large-size herds and flocks coupled with pastoralism practices may also play an important role in regions with limited resources.

Accordingly, national brucellosis control programs combined with mass vaccine campaigns were established in Iraq in 1996 and were continued after the war in 2003, with four vaccination campaigns introduced (2.1 million doses of Rev.1 vaccine and S19 doses of vaccine, types of vaccine used). Unfortunately, the campaigns stopped in 2010 [22].

Implementing different control measures in specific locations has the potential to maximize public health benefits while minimizing the spread of infection to areas with lower or no prevalence. However, effective implementation requires the involvement of all authorities in a “One-Health” approach.

An essential aspect is the need for vaccination against brucellosis in domestic ruminants. In certain epidemiological situations, vaccination can serve as a crucial starting point to enhance animal health, ensure food safety, and alleviate the economic implications associated with the presence and spread of the disease. Identifying the areas and animal species in which the adoption of vaccination programs may have long-term economic benefits is of utmost importance. Controlling and eliminating livestock brucellosis is crucial for reducing the disease in humans, and these actions are falling under veterinary responsibility [31].

The FAO/WHO report on *B. melitensis* in Eurasia and the Middle East [32] suggests zoning/compartmentalization within a country as a generic disease control measure applicable to *B. melitensis* control. However, effective compartmentalization necessitates a series of biosecurity measures, which may be challenging to implement in Iraq due to the intensity of non-regulated animal movements.

Brucellosis is also a growing global One-Health issue due to the increase in international commodity exchange, facilitating the spread of the infection from endemic areas to other parts of the world. However, it is understandable that even setting up a dataset like the one presented in this study may be very difficult, especially if we consider the social and economic context of the country concerned. Further studies are needed to better investigate the management and control strategies implemented in different regions of the country, as well as to expand the collection of isolates, to include strains obtained from more governorates, and correlate the findings with farm general information (e.g., management, size, location, climate, and infection history).

## 5. Conclusions

The aim of this study was to place *B. melitensis* strains in their position on the phylogenetic tree to compare them with other strains outside of the Iraqi region.

In the areas under study, the focus should be put on significant public health preventive interventions, such as livestock vaccination complemented by the development of related infrastructures. Robust campaigns targeting the general population to raise awareness about the disease are also crucial. Additionally, effective border control measures and the establishment of a regulatory framework for the control of this disease are essential. Collaborative efforts between regions, involving the exchange information, knowledge, and strategies for infectious disease prevention and control, are imperative for a comprehensive and unified approach across the country.

This study focused on the *B. melitensis* species, and the genomic approach allowed for typing by placing the sequenced strains within the context of thousands of isolates collected worldwide and genetically characterized over the last 10 years. This is an essential strategy to maintain correct epidemiological surveillance of brucellosis.

Furthermore, the control of brucellosis in Iraq requires a robust disease surveillance program managed collaboratively by the veterinary and health authorities to increase awareness among local health professionals within a “One-Health” framework, which will significantly enhance the diagnosis and management of brucellosis cases, contributing to the overall control and eradication of this disease.

## Figures and Tables

**Table 1:** *B. melitensis* strains analyzed in this study during the 2015–2017 period.

Strain ID	Governorates	Host	Collection Year
Iraq_2223-3	Karbala	Sheep	2017
Iraq_2223-6	Karbala	Sheep	2016
Iraq_2223-9	Baghdad	Cattle	2015
Iraq_2223-10	Karbala	Sheep	2017
Iraq_2223-11	Baghdad	Sheep	2015
Iraq_2223-12	Maysan	Sheep	2015
Iraq_2223-13	Wasit	Cattle	2015
Iraq_2223-19	Baghdad	Buffalo	2017
Iraq_2223-24	Baghdad	Sheep	2015
Iraq_2223-26	Babil	Sheep	2015
Iraq_2223-28	Diyala	Sheep	2015
Iraq_2223-35	Babil	Cattle	2015
Iraq_2223-39	Babil	Sheep	2017
Iraq_2223-40	Wasit	Sheep	2015
Iraq_2223-43	Wasit	Cattle	2015
Iraq_2223-47	Maysan	Cattle	2015
Iraq_2223-57	Babil	Sheep	2015
Iraq_2223-58	Babil	Sheep	2015
Iraq_2223-60	Baghdad	Sheep	2015
Iraq_2223-61	Maysan	Cattle	2015
Iraq_2223-66	Babil	Cattle	2017
Iraq_2223-75	Babil	Sheep	2017
Iraq_2223-76	Baghdad	Sheep	2015
Iraq_2223-78	Wasit	Cattle	2015
Iraq_2223-85	Babil	Sheep	2015
Iraq_2223-96	Karbala	Sheep	2017
Iraq_2223-98	Wasit	Sheep	2015
Iraq_2223-100	Wasit	Sheep	2015
Iraq_2223-102	Maysan	Cattle	2016
Iraq_2223-106	Karbala	Sheep	2016
Iraq_2223-108	Karbala	Sheep	2015
Iraq_2223-114	Wasit	Cattle	2015
Iraq_2223-117	Maysan	Cattle	2015
Iraq_2223-121	Wasit	Sheep	2015
Iraq_2223-122	Wasit	Cattle	2015

**Table 2:** *B. melitensis* strains analyzed in this study.

Strain ID	Governorates	Host	MLVA-16	MLVA Profile	Cg MLST Profile
Iraq_2223-3	Karbala	Sheep	1-5-3-13-3-2-3-2-4-40-8-8-4-3-15-4	9	9
Iraq_2223-6	Karbala	Sheep	1-5-3-13-3-2-3-2-4-40-8-5-4-3-16-4	6	8
Iraq_2223-9	Baghdad	Cattle	1-5-3-13-3-2-3-2-4-40-8-4-4-3-6-4	11	NA*
Iraq_2223-10	Karbala	Sheep	1-5-3-13-3-2-3-2-4-40-8-8-4-3-15-4	9	9
Iraq_2223-11	Baghdad	Sheep	1-5-3-13-3-2-3-2-4-40-8-4-4-3-15-4	1	5
Iraq_2223-12	Maysan	Sheep	1-5-3-13-3-2-3-2-4-40-8-4-4-3-16-4	2	11
Iraq_2223-13	Wasit	Cattle	1-5-3-13-3-2-3-2-4-40-8-4-4-3-19-4	4	4
Iraq_2223-19	Baghdad	Buffalo	1-5-3-13-3-2-3-2-4-40-8-4-4-3-9-4	12	NA*
Iraq_2223-24	Baghdad	Sheep	1-5-3-13-3-2-3-2-4-40-8-5-4-3-14-4	5	1
Iraq_2223-26	Babil	Sheep	1-5-3-13-3-2-3-2-4-40-8-4-4-3-15-4	1	7
Iraq_2223-28	Diyala	Sheep	1-5-3-13-3-2-3-2-4-40-8-4-4-3-15-4	1	NA*
Iraq_2223-35	Babil	Cattle	1-5-3-13-3-2-3-2-4-40-8-4-4-3-15-4	1	6
Iraq_2223-39	Babil	Sheep	1-5-3-13-3-2-3-2-4-40-8-8-4-3-15-4	9	9
Iraq_2223-40	Wasit	Sheep	1-5-3-13-3-2-3-2-4-40-8-4-4-3-19-4	4	NA*
Iraq_2223-43	Wasit	Cattle	1-5-3-13-3-2-3-2-4-40-8-4-4-3-19-4	4	4
Iraq_2223-47	Maysan	Cattle	1-5-3-13-3-2-3-2-4-40-8-8-4-3-15-4	9	9
Iraq_2223-57	Babil	Sheep	1-5-3-13-3-2-3-2-4-40-8-4-4-3-15-4	1	6
Iraq_2223-58	Babil	Sheep	1-5-3-13-3-2-3-2-4-40-8-4-4-3-15-4	1	7
Iraq_2223-60	Baghdad	Sheep	1-5-3-13-3-2-3-2-4-40-8-8-4-3-16-4	10	11
Iraq_2223-61	Maysan	Cattle	1-5-3-13-3-2-3-2-4-40-8-8-4-3-14-4	8	3
Iraq_2223-66	Babil	Cattle	1-5-3-13-3-2-3-2-4-40-8-4-4-3-15-4	1	NA*
Iraq_2223-75	Babil	Sheep	1-5-3-13-3-2-3-2-4-40-8-4-4-3-18-4	3	NA*
Iraq_2223-76	Baghdad	Sheep	1-5-3-13-3-2-3-2-4-40-8-4-4-3-15-4	1	5
Iraq_2223-78	Wasit	Cattle	1-5-3-13-3-2-3-2-4-40-8-5-4-3-19-4	7	4
Iraq_2223-85	Babil	Sheep	1-5-3-13-3-2-3-2-4-40-8-4-4-3-15-4	1	NA*
Iraq_2223-96	Karbala	Sheep	1-5-3-13-3-2-3-2-4-40-8-8-4-3-15-4	9	NA*
Iraq_2223-98	Wasit	Sheep	1-5-3-13-3-2-3-2-4-40-8-8-4-3-15-4	9	10
Iraq_2223-100	Wasit	Sheep	1-5-3-13-3-2-3-2-4-40-8-8-4-3-15-4	9	9
Iraq_2223-102	Maysan	Cattle	1-5-3-13-3-2-3-2-4-40-8-4-4-3-15-4	1	9
Iraq_2223-106	Karbala	Sheep	1-5-3-13-3-2-3-2-4-40-8-5-4-3-16-4	6	8
Iraq_2223-108	Karbala	Sheep	1-5-3-13-3-2-3-2-4-40-8-4-4-3-19-4	4	NA*
Iraq_2223-114	Wasit	Cattle	1-5-3-13-3-2-3-2-4-40-8-4-4-3-19-4	4	NA*
Iraq_2223-117	Maysan	Cattle	1-5-3-13-3-2-3-2-4-40-8-5-4-3-14-4	5	2
Iraq_2223-121	Wasit	Sheep	1-5-3-13-3-2-3-2-4-40-8-4-4-3-19-4	4	4
Iraq_2223-122	Wasit	Cattle	1-5-3-13-3-2-3-2-4-40-8-4-4-3-19-4	4	4

NA*—Not Available.

## Data Availability

The raw sequencing data generated in this study are available under NCBI Bioproject ac-cession no. PRJNA1070976 and the remaining data are contained within the article.
